# Fahr’s syndrome as the initial imaging characteristics of MELAS syndrome with a possible seizure activity and cardiac arrest: a case report

**DOI:** 10.3389/fgene.2024.1393158

**Published:** 2024-08-12

**Authors:** Yan Zheng, Haohao Wu, Meng Zhang, Baogang Huang, Junsu Yang, Chuan Liu, Hanmin Wang, Kang Du

**Affiliations:** ^1^ Department of Neurology, Qujing First People’s Hospital, Qujing, Yunnan, China; ^2^ Teaching and Research Office of Internal Medicine, Qujing Medical College, Qujing, Yunnan, China; ^3^ Department of Urology, Qujing First People’s Hospital, Qujing, Yunnan, China; ^4^ Department of Endocrinology and Metabolism, Qujing First People's Hospital, Qujing, Yunnan, China

**Keywords:** MELAS syndrome, Fahr’s syndrome, near-SUDEP, LPD, UPJO

## Abstract

This study reported a case of MELAS syndrome presenting as the initial imaging characteristics of Fahr’s syndrome with “near” sudden unexpected death in epilepsy (SUDEP) and lateralized periodic discharges (LPD). The patient, a young boy, experienced loss of consciousness 2 days prior, which was followed by two limb and facial convulsions. He was later found in cardiac arrest during hospitalization, but regained consciousness gradually after receiving cardiopulmonary resuscitation and tracheal intubation. The patient exhibited short stature, intellectual disability, poor sports abilities, and academic performance since childhood, but had no family history. Emergency head computed tomography (CT) revealed high density calcification in bilateral caudate nucleus, lentiform nucleus, thalamus, and dentate nucleus with evidence of an acute process. The patient was transferred to the neurology department where he continued to recover consciousness, though he experienced dysarthria, left limb hemiplegia, and hemiparesthesia. Changes in head magnetic resonance imaging (MRI) findings were noted at admission, 1 month later, and 6 months later. LPD were observed in his video electroencephalogram. The CT urography indicated a narrow left ureteropelvic junction with left hydronephrosis, which was suggestive of ureteropelvic junction obstruction. Ultimately, a diagnosis of near-SUDEP was suspected in this patient, indicating a rare case of MELAS syndrome with near-SUDEP and LPD. The gene tests results revealed the presence of the mitochondrial DNA A3243G mutation, leading to the final diagnosis of MELAS syndrome. This case expands the clinical disease spectrum of the MELAS syndrome.

## Introduction

Mitochondrial encephalomyopathy, lactic acidosis, and stroke-like episodes (MELAS) syndrome is a multi-organ disease with a wide range of manifestations, including stroke-like episodes, dementia, epilepsy, lactatemia, myopathy, recurrent headaches, hearing impairment, diabetes, and short stature. The most common mutation associated with MELAS syndrome is the m.3243 A>G mutation of the MT-TL1 gene, which encodes mitochondrial tRNALeu (El-Hattab et al., 2015). “Fahr’s syndrome” first appeared in literature in 1982 to describe the constellation of neuropsychiatric features and calcification, and the term has been used in the literature to indicate cases of secondary basal ganglia calcification (Saleem et al., 2013; Batla et al., 2017). The most common secondary cause of Fahr’s syndrome is parathyroid disease, although mitochondrial disease can also exhibit the imaging manifestations associated with this condition (Etcharry-Bouyx et al., 1995; Saleem et al., 2013). However, Fahr’s syndrome as an initial imaging feature is rarely reported in MELAS (Jimenez-Ruiz et al., 2018). Sudden unexpected death in epilepsy (SUDEP) is a sudden seizure-related death outcome, and is also the leading cause of epilepsy-related death in children and adults (Whitney et al., 2023a). In this report, we present a case of MELAS syndrome with Fahr’s syndrome as the initial imaging feature, lateralized periodic discharges (LPD) in the patient’s electroencephalogram (EEG) and a suspicious diagnosis of “near-SUDEP”.

## Case presentation

**FIGURE 1:**
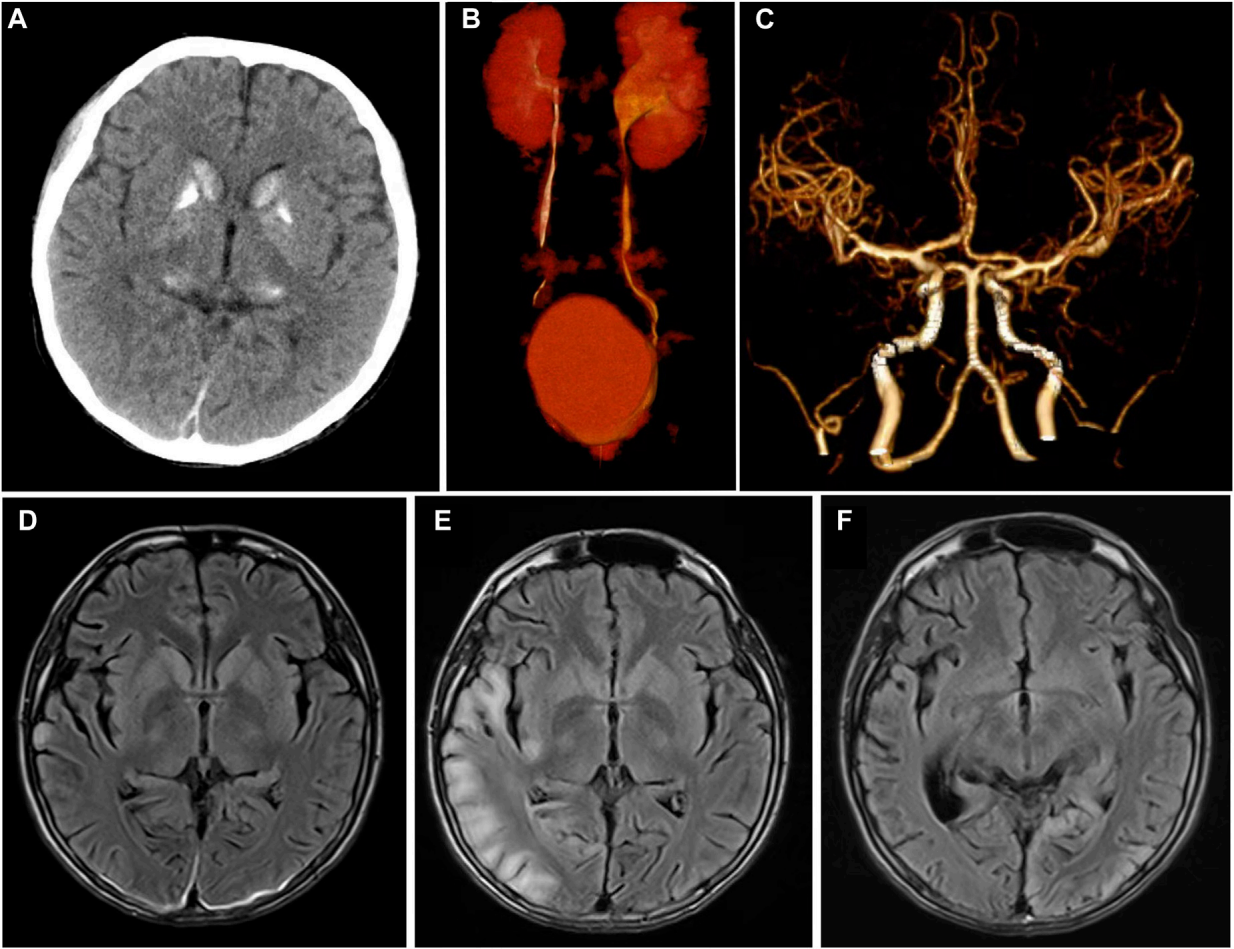
Imaging characteristics at admission and follow-up of this patient. **(A)** Symmetrical calcification of bilateral basal ganglia and thalamus was showed in head CT at admission; **(B)** CT urography showed that the left ureteropelvic junction was narrow with left hydronephrosis; **(C)** No abnormality in the head CTA. **(D–F)** Showed the cranial MRI T2flair characteristics of this patient at admission, after 1 month and 6 months of follow-up. After admission, only the right occipital cortex showed laminated high signal **(D)**. One month later, the patient showed typical high signal intensity area in the right occipital lobe **(E)**. Six months later, the right occipital lobe lesion disappeared and transferred to the left occipital cortex **(F)**.

An 18-year-old boy with a history of left hydronephrosis surgery 3 years ago was admitted to our emergency department after experiencing acute onset of neurological symptoms. The patient lost consciousness and exhibited salivation and incontinence 2 days prior, followed by two limb and face convulsions accompanied by staring eyes and hand abduction, each lasting 10–20 s, considering the possibility of cluster seizures. Additionally, he experienced a cardiac arrest during the seizures, but his consciousness gradually returned after receiving cardiopulmonary resuscitation and tracheal intubation. Since childhood, the boy has had short stature, intellectual disability, poor motor skills, and academic performance, with no family history of similar conditions. Emergency head computed tomography (CT) and computerized tomography angiography (CTA) scans revealed high density calcification in the bilateral caudate nucleus, lentiform nucleus, thalamus, and dentate nucleus, but no obvious intracranial stenosis or acute processes were observed (Figures 1A, C). The patient was subsequently transferred to the neurology department, where he gradually recovered consciousness but has persistent dysarthria, left limb hemiplegia, and hemiparesthesia.

He received continuous vasopressors to maintain blood pressure. And the physical examination was performed after cardiopulmonary resuscitation and tracheal intubation, it was noted that his BMI was 12.5, he exhibited moderate dysarthria, poor response, and hearing impairment in both ears. The muscle strength of the proximal of his left upper limb was Grade 2, while the distal was Grade 4. The muscle strength of his left lower limb was Grade 4, the muscle strength of his right limb was Grade 5, and muscle tension in his limbs was reduced. Additionally, there was diffuse muscle atrophy in the limbs, and reduced pain on the left side. Tendon reflexes in his limbs were symmetrically weakened, and no other positive signs were found or he could not cooperate with other physical examinations. Furthermore, blood tests revealed mild to moderate anemia [HGB 94 g/L (reference range 130–175)], elevated levels of lactate dehydrogenase at 881 U/L (reference range 120–250), creatine kinase at 1,467 (reference range 0–200), troponin I at 0.321 ug/mL (reference range 0–0.056), myoglobin at 2635 ng/mL (reference range 10–92), Nt-proBNP1393 pg/mL (reference range 0–125), and C-reactive protein at 92.7 g/L (reference range 0–10). His blood lactic acid levels were elevated at 8.3 mmol/L (reference range 0.5–2.2 mmol/L), and fasting blood glucose levels were normal at 4.9 mmol/L. Additionally, pituitary prolactin levels were below the reference range at 72.77 mIU/L (reference range 87–392), insulin-like growth-binding protein-3 at 1.72 ug/mL (reference range 3.1–7.9), growth hormone at 7.66 ug/L (reference range 0–3), total thyroxine at 36.07 nmol/L (reference range 55–160), Free T3 at 1.47 pmol/L (reference range 3.21–5.76), and his serum sodium levels were within normal limits at 143.3 mmol/L (reference range 137–147). Parathyroid hormone levels were slightly low at 11.5 (reference range 12–65) pg/mL.

An electrocardiogram revealed sinus tachycardia and Wolff-Parkinson-White (WPW) syndrome type A. Urinary CT and CT urography suggested a narrow left ureteropelvic junction with left hydronephrosis, which was confirmed by urologists as ureteropelvic junction obstruction (UPJO) (Figure 1B). The chest CT showed pneumonia in lower lobe of both lungs. Head magnetic resonance imaging (MRI) showed symmetric abnormal signals in bilateral basal ganglia (Figure 1D).

**FIGURE 2:**
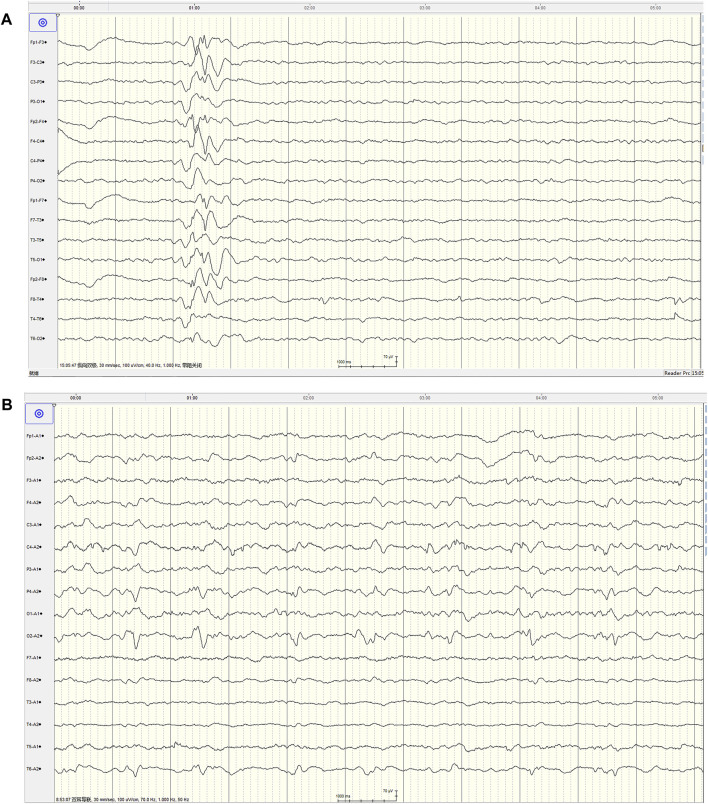
The video EEG characteristics at the first admission and 1-month follow-up of this patient. **(A)** Showed typical spine and slow wave distribution (00:15:07), mainly in area of Fp2-F4 and Fp2-F8, and conducted to Fp1-F3 at 222 m, and no clinical onset of the patient was observed (at admission) (Montage:Bipolar longitudinal connection, TR:30 mm/s, Sensitivity:100 μV/cm, HF:40 Hz, LF:1.0 Hz). **(B)** Showed lateralized periodic discharges (LPD) composed of sharp slow wave, interval cycle: 1.17–1.58 s, mainly in area of O1, no clinical onset of the patient was observed (1 month later) (Montage:Earlobe reference, TR:30 mm/s, Sensitivity:100 μV/cm, HF:70 Hz, LF:1.0 Hz).

The patient completed the lumbar puncture examination, and the results of cerebrospinal fluid showed that the nucleated cells was 4 × 10^6^/L, protein was 0.6 g/L (reference range <0.5), and there were no abnormal for others. Continuous EEG monitoring showed scattered in the low-medium-high-ultra-high amplitude spine waves, spine slow waves (Figure 2A). Treatment with levetiracetam for epilepsy, norepinephrine suppression, assisted ventilation, anti-infection measures, and symptomatic support resulted in gradual improvement of the patient’s consciousness and spontaneous breathing. However, lingering symptoms such as dizziness, left limb hemiplegia, left limb hemiparesthesia, dysarthria, and occasional left limb twitching persisted. Due to the patient’s short stature, mental abnormalities, and intolerance to motor activities, we made a suspicion of mitochondrial disease in light of the multisystem involvement. A hearing examination revealed a mean hearing threshold of 65 dB in the right ear and 62 dB in the left ear, indicative of sensorineural hearing loss.

The nerve conduction study indicated that the conduction velocity of the left tibial nerve and bilateral sural nerve was slightly to moderately slowed down, while the residual sensory nerves and motor nerves conduction examination showed no obvious abnormalities. Due to the possibility of mitochondrial diseases, mitochondrial gene testing was performed. The results revealed the presence of the mitochondrial DNA A3243G point mutation, leading to the final diagnosis of MELAS syndrome. The patient was treated with levetiracetam, arginine hydrochloride, coenzyme Q10, and aldibenquinone, which resulted in improvement and eventual discharge. However, 1 month after discharge, the patient experienced intermittent seizures in the left upper limb following discontinuation of levetiracetam 3 days before he was readmitted to the hospital, lasting 10 s each time. There were no new neurological signs observed. A 1-month follow-up head MRI was performed, which revealed abnormal signals in the right temporoccipital parietal cortex (Figure 1E). The patient adhered to regular medication and the condition remained stable. After 6 months, the patient returned for a follow-up visit and a repeat head MRI scan showed that the lesions transferred to the left occipital cortex (Figure 1F), which showed the imaging characteristics of typical MELAS syndrome. The second follow-up of the video EEG indicated a typical pattern of LPD appeared in the occipital area (Figure 2B). Anti-epileptic drugs continued, and no seizures occurred. The patient was improved upon discharge with recommendations to follow up with outpatient neurology.

## Discussion

MELAS syndrome is the most common type of mitochondrial encephalomyopathy, which is a maternally inherited disease. There are also sporadic cases, with the onset age of most patients ranging from 2 to 31 years old (El-Hattab et al., 2015). The patient in this case had short stature since childhood, and his brother, mother, and father showed no similar manifestations. They refused to undergo genetic testing. The clinical manifestations of this patient included stroke-like episodes, epilepsy, short stature, cognitive impairment, limb weakness, sensorineural deafness, and endocrine system disorders. The clinical manifestations of MELAS are diverse and lack specificity (Branch et al., 2020). Among them, UPJO was a common pediatric congenital urinary malformation that had not been reported in previous MELAS patients to our knowledge. The main cause of UPJO was the replacement of loose and irregular ureteropelvic junction (UPJ) spiral-shaped muscle fibers by abnormal longitudinal muscle fibers or fibrous tissue. This led to the disappearance of normal peristaltic waves at the UPJ and restricted urine flow from the renal pelvis into the proximal ureter. It was unclear whether ureteral stricture was directly related to MELAS or simply a coincidence. And we are inclined to believe that there may be a coincidence.

The intracranial lesions associated with MELAS syndrome were predominantly located in the cortex and subcortical white matter of the occipital, parietal, and temporal lobes. Interestingly, these areas did not align with the blood supply distribution of the cerebral vessels (Mascalchi et al., 2018). The head CT of MELAS syndrome patients shows bilateral symmetrical calcification in the basal ganglia, with imaging features resembling Fahr’s syndrome present in about 13% of cases (Donzuso et al., 2019). In this case, the initial imaging findings revealed symmetrical high density in the basal ganglia without typical MRI findings. This suggests that patients exhibiting isolated imaging features of Fahr’s syndrome should be vigilant about the potential presence of MELAS syndrome. Additionally, it was important to conduct follow-up visits and repeats of head MRI scan in patients suspected of MELAS syndrome (Danhua et al., 2014).

Periodic discharges are defined as abnormal discharges that stand out from the background activity, occurring at roughly equal intervals and lasting for at least six cycles. These discharges are categorized into generalized periodic discharges (GPD) and LPD (Hirsch et al., 2021). Periodic discharges are a relatively severe EEG abnormal discharge that is rare. It is more commonly seen in cases of acute structural brain injury (Sakathevan et al., 2022), presenting with periodic discharges. However, LPD in MELAS syndrome is rarely reported. Lin and Drislane (2018) reported seven cases of MELAS syndrome with EEG data, but only one case showed LPD.

Our patient also exhibited a typical short period of LPD during the second EEG monitoring. The two EEG recordings clearly demonstrated the changing process of abnormal discharges. In the initial visit of EEG, only an irregular slow wave distribution in the anterior head was noted, but during the second visit, a typical LPD consisting of sharp and slow waves emerged in the occipital area. This demonstrated a clear changing process with changes in morphology and position. This EEG evolution process of MELAS was first reported as early as 2003 (Iizuka et al., 2003), one patient displayed a Focal sharp-LPD-Focal sharp changing pattern, while two other patients exhibited an LPD-Focal sharp changing pattern. Our patient, on the other hand, showed a spike and wave complex-LPD changing pattern. We believed this pattern was linked to the progression of the disease and the changing of the lesions. And LPD may not be present in the early stages but could develop as the disease advances, possibly disappearing in later stages or when the disease stabilizes. Unfortunately, we did not have EEG data for our patient in the late stage of the disease. Additionally, we observed that the spike wave did not align with the lesion location (temporal-occipital) in the initial EEG recording. However, the changing of LPD (occipital area) corresponded with the lesion location (temporal and occipital), indicating that LPD was a more accurate marker for localizing lesions in patients.

In recent years, more attention has been focused on SUDEP (Whitney et al., 2023a), (Burneo, 2019). SUDEP is more common among young people aged 20–45 years old. It often occurs following tonic and clonic seizures, particularly at night. While the exact mechanism of onset for SUDEP is not fully understood, fatal arrhythmias are widely recognized as occurring during seizures. Additionally, certain genetic diseases affecting heart conduction and neuronal excitability may also be risk factors for SUDEP (Devinsky et al., 2016). Whitney et al. (2023b) have found that, there were pathogenic variants found in epilepsy-related genes and cardiac genes in the autopsy cases of SUDEP, prompting scholars to focus on the genetic mechanism of this condition. MELAS syndrome is a genetic disease known to cause cardiac conduction disorders (Cosma et al., 2023). Patients with the A3243G mutation may exhibit a wide range of cardiac disease features, including abnormalities in conduction and the myocardium (Finsterer, 2009). Therefore, SUDEP/near-SUDEP could theoretically occur in MELAS syndrome patients with epilepsy, although there have been no reported case.

The patient’s electrocardiogram revealed sinus tachycardia, a short PR interval, and WPW syndrome type A and we suspected that the patient had previous arrhythmias, which may have contributed to the fatal arrhythmia during the seizure, possibly due to autonomic dysfunction (Sproule et al., 2007). However, we do not believe the cardiac arrest was due to the WPW syndrome. Unfortunately, due to the lack of continuous electrocardiogram, EEG, and respiratory monitoring at the time of the “near-SUDEP” event, which was a critical situation, it is difficult to determine whether the patient’s “near-SUDEP” was caused by a cardiac issue or directly by abnormal brain electrical activity affecting the cardiac and respiratory centers. This is a regrettable situation for us, as there are various explanations for SUDEP, with cardiac conduction issues being just one possibility and a speculative one at that. Further exploration is needed to understand the relationship between the A3243G mutation and SUDEP. If the cardiac arrest in our case was triggered by seizure activity, this suggests that seizure activity in MELAS syndrome may put individuals at risk for SUDEP. And the mechanism for seizure-associated cardiac arrest may resemble that for established SUDEP or near-SUDEP cases. Thus, we recommend closely monitoring MELAS syndrome patients with both arrhythmias and seizures in order to identify and possibly correct risk factors for SUDEP/near-SUDEP. ECG and continuous bedside EEG monitoring, especially at night, are crucial for early detection and intervention (Devinsky et al., 2016), and controlling seizures and arrhythmias is an important measure to prevent SUDEP/near-SUDEP in patients with this condition.

## Data Availability

The datasets presented in this article are not readily available because of ethical and privacy restrictions. Requests to access the datasets should be directed to the corresponding author.

## References

[B1] Batla A. Tai X. Y. Schottlaender L. Erro R. Balint B. Bhatia K. P. (2017). Deconstructing Fahr's disease/syndrome of brain calcification in the era of new genes. Park. Relat. Disord. 37, 1–10. 10.1016/j.parkreldis.2016.12.024 28162874

[B2] Branch BMAR Neuromuscular D.NBBM Group CMDC (2020). Chinese expert consensus on diagnosis and treatment of mitochondrial encephalomyopathy with lactic acidosis and stroke-like episodes. Chin. J. Neurology 53, 171–178. 10.3760/cma.j.issn.1006-7876.2020.03.003

[B3] Burneo J. G. (2019). SUDEP: let's talk about it. Neurology 93, 93–94. 10.1212/WNL.0000000000007771 31217260

[B4] Cosma J. Russo A. Schino S. Belli M. Mango R. Chiricolo G. (2023). Acute myocardial infarction in a patient with MELAS syndrome: a possible link? Minerva Cardiol. Angiol. 71, 374–380. 10.23736/S2724-5683.22.06021-5 35767235

[B5] Danhua Z. Zhaoxia W. Lei Y. Jiangxi X. Sheng X. Yun Y. (2014). Dynamic evolution of brain magnetic resonance imaging findings in patients with mitochondrial myopathy, encephalopathy, lactic acidosis, and stroke-like episodes syndrome. Chin. J. Neurology 47, 229–231. 10.3760/cma.j.issn.1006-7876.2014.04.004

[B6] Devinsky O. Hesdorffer D. C. Thurman D. J. Lhatoo S. Richerson G. (2016). Sudden unexpected death in epilepsy: epidemiology, mechanisms, and prevention. Lancet Neurol. 15, 1075–1088. 10.1016/S1474-4422(16)30158-2 27571159

[B7] Donzuso G. Mostile G. Nicoletti A. Zappia M. (2019). Basal ganglia calcifications (Fahr's syndrome): related conditions and clinical features. Neurol. Sci. 40, 2251–2263. 10.1007/s10072-019-03998-x 31267306 PMC6817747

[B8] El-Hattab A. W. Adesina A. M. Jones J. Scaglia F. (2015). MELAS syndrome: clinical manifestations, pathogenesis, and treatment options. Mol. Genet. Metab. 116, 4–12. 10.1016/j.ymgme.2015.06.004 26095523

[B9] Etcharry-Bouyx F. Ceccaldi M. Poncet M. Pellissier J. F. (1995). Fahr's disease and mitochondrial myopathy. Rev. Neurol. Paris. 151 (151), 731–733. 8787104

[B10] Finsterer J. (2009). Manifestations of the mitochondrial A3243G mutation. Int. J. Cardiol. 137, 60–62. 10.1016/j.ijcard.2008.04.089 18662836

[B11] Hirsch L. J. Fong M. Leitinger M. LaRoche S. M. Beniczky S. Abend N. S. (2021). American clinical neurophysiology society's standardized critical care EEG terminology: 2021 version. J. Clin. Neurophysiol. 38, 1–29. 10.1097/WNP.0000000000000806 33475321 PMC8135051

[B12] Iizuka T. Sakai F. Kan S. Suzuki N. (2003). Slowly progressive spread of the stroke-like lesions in MELAS. Neurology 61, 1238–1244. 10.1212/01.wnl.0000091888.26232.fe 14610127

[B13] Jimenez-Ruiz A. Cardenas-Saenz O. Ruiz-Sandoval J. L. (2018). Symmetrical and bilateral basal ganglia calcification. Case series and literature review. Gac. Med. Mex. 154, 258–262. 10.24875/GMM.18002406 29733071

[B14] Lin L. Drislane F. W. (2018). Lateralized periodic discharges: a literature review. J. Clin. Neurophysiol. 35, 189–198. 10.1097/WNP.0000000000000448 29718828

[B15] Mascalchi M. Montomoli M. Guerrini R. (2018). Neuroimaging in mitochondrial disorders. Essays Biochem. 62, 409–421. 10.1042/EBC20170109 30030366

[B16] Sakathevan J. Somasundaram K. Chinyere S. C. Rodriguez-Vina C. Martin-Lopez D. (2022). Lateralized periodic discharges during remifentanil infusion. Clin. EEG Neurosci. 53, 143–147. 10.1177/15500594211010624 33900123

[B17] Saleem S. Aslam H. M. Anwar M. Anwar S. Saleem M. Saleem A. (2013). Fahr's syndrome: literature review of current evidence. Orphanet J. Rare Dis. 8, 156. 10.1186/1750-1172-8-156 24098952 PMC3853434

[B18] Sproule D. M. Kaufmann P. Engelstad K. Starc T. J. Hordof A. J. De Vivo D. C. (2007). Wolff-Parkinson-White syndrome in patients with MELAS. Arch. Neurol. 64, 1625–1627. 10.1001/archneur.64.11.1625 17998445

[B19] Whitney R. Jones K. C. Sharma S. RamachandranNair R. (2023a). SUDEP counseling: where do we stand?. Epilepsia 64, 1424–1431. 10.1111/epi.17617 37039574

[B20] Whitney R. Sharma S. Jones K. C. RamachandranNair R. (2023b). Genetics and SUDEP: challenges and future directions. Seizure 110, 188–193. 10.1016/j.seizure.2023.07.002 37413779

